# Human platelets generate phospholipid-esterified prostaglandins via cyclooxygenase-1 that are inhibited by low dose aspirin supplementation[S]

**DOI:** 10.1194/jlr.M041533

**Published:** 2013-11

**Authors:** Maceler Aldrovandi, Victoria J. Hammond, Helen Podmore, Martin Hornshaw, Stephen R. Clark, Lawrence J. Marnett, David A. Slatter, Robert C. Murphy, Peter W. Collins, Valerie B. O'Donnell

**Affiliations:** * Institute of Infection and Immunity, School of Medicine, Cardiff University; † Cardiff, United Kingdom ThermoFisher Scientific, Hemel Hempstead, United Kingdom; § Vanderbilt Institute of Chemical Biology, Center in Molecular Toxicology, Vanderbilt-Ingram Cancer Center, Nashville, TN; ** Department of Pharmacology, University of Colorado Denver, Aurora, CO

**Keywords:** Oxidized phospholipids, atherosclerosis, PGE_2_/D_2_-PEs

## Abstract

Oxidized phospholipids (oxPLs) generated nonenzymatically display pleiotropic biological actions in inflammation. Their generation by cellular cyclooxygenases (COXs) is currently unknown. To determine whether platelets generate prostaglandin (PG)-containing oxPLs, then characterize their structures and mechanisms of formation, we applied precursor scanning-tandem mass spectrometry to lipid extracts of agonist-activated human platelets. Thrombin, collagen, or ionophore activation stimulated generation of families of PGs comprising PGE_2_ and D_2_ attached to four phosphatidylethanolamine (PE) phospholipids (16:0p/, 18:1p/, 18:0p/, and 18:0a/). They formed within 2 to 5 min of activation in a calcium, phospholipase C, p38 MAP kinases, MEK1, cPLA_2_, and *src* tyrosine kinase-dependent manner (28.1 ± 2.3 pg/2 × 10^8^ platelets). Unlike free PGs, they remained cell associated, suggesting an autocrine mode of action. Their generation was inhibited by in vivo aspirin supplementation (75 mg/day) or in vitro COX-1 blockade. Inhibitors of fatty acyl reesterification blocked generation significantly, while purified COX-1 was unable to directly oxidize PE in vitro. This indicates that they form in platelets via rapid esterification of COX-1 derived PGE_2_/D_2_ into PE. In summary, COX-1 in human platelets acutely mediates membrane phospholipid oxidation via formation of PG-esterified PLs in response to pathophysiological agonists.

Prostaglandins (PGs) are a family of lipid signaling mediators generated by cyclooxygenase (COX) enzymes, COX-1 and -2. They play central roles in health, as well as in diseases including cancer and atherosclerosis. Until recently, they were considered to only exist as free acid mediators, secreted from cells to activate G-protein coupled receptors in a paracrine manner. In 2005, Kozak et al. (1, 2) showed prostaglandins E_2_ and D_2_ (PGE_2_, PGD_2_) were generated in macrophage cell lines from COX-2 oxidation of endogenous arachidonyl-glycerol (2-AG) and arachidonyl-ethanolamide (AEA). The products signal differently to free PGE_2_ and D_2_; for example, PGE_2_-G mobilizes calcium rapidly in a PGE_2_-independent manner, indicating they are chemically and functionally distinct from their free acid analogs (3, 4).

Oxidized phospholipids (oxPLs) were originally characterized as nonenzymatically-generated species present in atheromatous plaque that display potent immunomodulatory activities (5–7). Recent studies have indicated that they are also generated in a highly specific manner by cellular lipoxygenases (LOXs) in neutrophils, monocytes and platelets (8–14). Also, oxidized cholesteryl esters formed by 15-lipoxygenase in macrophages can transfer the oxidized fatty acyl group to phospholipids (15). However, oxPLs generated by COX isoforms have not been described. Furthermore, whereas COX-2 can oxidize AEA and 2-AG, the constitutive isoform COX-1 has not been shown to be a source of any esterified eicosanoids before. Herein, we used a targeted lipidomic approach to demonstrate that human platelets generate phospholipid (PL)-esterified PGE_2_ and PGD_2_ on agonist activation. Their synthesis is highly regulated, involving receptors and a number of key intracellular signaling pathways. Thus, oxPLs can be generated acutely via COX-1 and represent a new family of lipids from this important vascular signaling pathway.

## MATERIALS AND METHODS

### Materials

Lipid and fatty acid standards were purchased from Avanti Polar Lipids (Alabaster, AL) or Cayman Chemical (Cayman Islands). HPLC grade solvents were from Thermo Fisher Scientific (Hemel Hempstead, UK). Protease-activated receptor (PAR)-1 and -4 agonists were from Tocris Biosciences (Bristol, UK). Triacsin C, PGE_2_, PGD_2_, 8-iso-PGE_2_, and 11β-PGE_2_ were from Enzo Life Sciences (Exeter, UK). COX-1 inhibitor (Sc-560) PGE2-d4, PGD2-d4, and AA-d8 were from Cayman Chemical. Platelet signaling inhibitors [PP2, oleyloxyethylphosphocholine (OOEPC), bromoenol lactone (BEL), cytosolic phospholipase A_2α_ (cPA_2α_) inhibitor (N-((2S,4R)-4-(Biphenyl-2-ylmethyl-isobutyl-amino)-1-[2-(2,4-difluorobenzoyl)-benzoyl]pyrrolidin-2-ylmethyl}-3-[4-(2,4-dioxothiazolidin-5-ylidenemethyl)-phenyl]acrylamide, HCl), U73112, wortmannin, and p38 mitogen-activated protein kinase inhibitor] were from Calbiochem. All other reagents were from Sigma-Aldrich unless otherwise stated.

### Precursor LC-MS/MS

Lipid extracts were separated by reverse-phase HPLC using a Luna 3 μm C18 (2) 150 × 2 mm column (Phenomenex, Torrance, CA) with a gradient of 50–100% B over 10 min followed by 30 min at 100% B (A, methanol:acetonitrile:water, 1 mmol/L ammonium acetate, 60:20:20; B, methanol, 1 mmol/L ammonium acetate) with a flow rate of 200 μl.min^−1^. Settings were DP -140 V, CE -45 V. Spectra were acquired scanning Q1 from 650 to 950 atomic mass units (amu) over 5 s with Q3 set to *m/z* 351.2.

### Normal-phase HPLC of phospholipids

Phospholipids were separated according to headgroup using normal-phase HPLC, on a Spherisorb S5W 150 × 4.6 mm column (Waters Ltd. Herts, UK) with a gradient of 50 - 100% B over 25 min (A, hexane:propan-2-ol, 3:2; B, solvent A:water, 94.5:5.5) at a flow rate of 1.5 ml.min^−1^. Absorbance was monitored at 205 nm and products identified using a mixture of standard phospholipids (Sigma-Aldrich). Fractions were collected at 30 s intervals for subsequent analysis by direct injection ESI/MS/MS on a Sciex 4000 Q-Trap. This was performed by injecting 20 µl of each fraction under flow (1 ml.min^−1^) in methanol into the electrospray source, with specific multiple reaction monitoring (MRM) transitions monitored as parent of *m/z* 770.6, 796.6, 798.6 and 814.6 [M-H]^−^, fragmenting to daughter of *m/z* 351.2. Settings were DP -140 V, CE -45 V.

### Reverse-phase LC-MS/MS of PG isomers

Separation of 8-isoPGE_2_, 11β-PGE_2_, PGE_2_, and PGD_2_ was conducted using a C18 Spherisorb ODS2, 5 μm, 150 × 4.6 mm column (Waters, UK). The solvent system was composed of 0.1% formic acid in water (solvent A) and 0.1% formic acid in acetonitrile (solvent B), with flow rate 1 ml.min^−1 (16)^. Solvent B was increased from 20% to 42.5% over 50 min, then increased to 90% over 10.5 min, held for 5 min, then returned to 20% over 1 min. Equilibration time between runs was 14 min. Settings were DP -50 V, CE -26 V. The following transitions were monitored: *m/z* 351.2 → 271 (8-isoPGE_2_, 11β-PGE_2_, PGE_2_ and PGD_2_), *m/z* 355.2 → 275.3 (PGE_2_-d4 and PGD_2_-d4).

### Reverse-phase LC-MS/MS of esterified eicosanoids

For analysis of esterified prostaglandins in MRM mode, lipid extracts were separated by reverse-phase HPLC using a Luna 3 μm C18 (2) 150 mm × 2 mm column (Phenomenex) with a gradient of 50–100% B over 10 min followed by 30 min at 100% B (A, methanol/acetonitrile/water, 1 mmol/L ammonium acetate, at 60:20:20; B, methanol, 1 mmol/L ammonium acetate) with flow rate 200 μl min ^–1^. MS was carried out using a Sciex 4000 Q-Trap, using DP -140 V, CE -45 V. Lipids were monitored as parent *m/z* to daughter (*m/z* 351.2 or 271.2, as appropriate), with dwell time 200 ms. For analysis of lipids by full scan using accurate mass, lipids were separated by reverse-phase HPLC coupled to an Orbitrap Velos, on a C18 Hypersil Gold, 1.9 µm, 100 × 2.1 mm column using a gradient of 50–100% B over 10 min followed by 15 min at 100% B, then resetting to starting conditions over 5 min (A, methanol/acetonitrile/water, 1 mmol/L ammonium acetate, 60:20:20; B, methanol, 1 mmol/L ammonium acetate) with a flow rate of 200 μl min ^–1^. Analysis was performed using heated ESI in negative ion mode at sheath, auxiliary, and sweep gas flows of 30, 10, and 0, respectively. The capillary and source heater temperatures were set to 275 and 250°C, respectively. Resolving power of 30,000 in FTMS mode was used. Negative MS/MS spectra were acquired using higher energy collision-induced-dissociation. Data-dependent MS (3) of *m/z* 351 was carried out in ITMS mode on the LTQ in negative mode.

### Isolation and activation of human platelets

All blood donations were approved by the Cardiff University School of Medicine Ethics Committee, were with informed consent (SMREC 12/37, SMREC 12/10), and were according to the Declaration of Helsinki. For studies on isolated platelets, whole blood was collected from healthy volunteers free from nonsteroidal anti-inflammatory drugs for at least 14 days into acid-citrate-dextrose (ACD; 85 mmol/L trisodium citrate, 65 mmol/L citric acid, 100 mmol/L glucose) (blood:ACD, 8.1:1.9, v/v) and centrifuged at 250 *g* for 10 min at room temperature. Platelet-rich plasma was collected and centrifuged at 900 *g* for 10 min, and the pellet resuspended in Tyrode's buffer (134 mmol/L NaCl, 12 mmol/L NaHCO_3_, 2.9 mmol/L KCl, 0.34 mmol/L Na_2_HPO_4_, 1.0 mmol/L MgCl_2_,10 mmol/L Hepes, 5 mmol/L glucose, pH 7.4) containing ACD (9:1, v/v). Platelets were centrifuged at 800 *g* for 10 min then resuspended in Tyrode's buffer at 2 × 10^8^.ml^−1^. Platelets were activated at 37°C in the presence of 1 mmol/L CaCl_2_ for varying times, with 0.2 unit.ml^−1^ thrombin, 10 μg/ml collagen, 10 μmol/L A23187, 20 μmol/L TFLLR-NH_2_, or 150 μmol/L AY-NH_2_ before lipid extraction as below. Experiments involving signaling inhibitors (1 mmol/L aspirin, 1 µmol/L SC-560, 10 µmol/L indomethacin, 2 µmol/L OOEPC, 50 nmol/L BEL, 50 nmol/L cPLA_2α_i, 75 µM thimerosal, 7 μM triascin C, 1 mM EGTA, 10 µM 1,2-bis-(*o*-aminophenoxy) ethane-*N,N,N′,N′-*tetraacetic acid tetrakis-acetoxymethyl ester (BAPTA/AM), 100 nM wortmannin, 100 nmol/L Gö 6850, 50 μmol/L PD98059, 50 µmol/L PP2, 100 nmol/L p38 mitogen-activated protein kinase inhibitor, and 5 µM U-73122) included a 10 min preincubation at room temperature. In some experiments, calcium was omitted from buffers. For separation of cells from microparticles, platelets were centrifuged at 970 *g* for 5 min then supernatants respun at 16,060 *g* for 5 min. For aspirin supplementation, blood samples were first obtained following a 14-day NSAID-free period for baseline determinations of eicosanoids. Subjects were administered 75 mg/day aspirin for 7 days then provided a second blood sample. Platelets were isolated and activated in vitro using 0.2 U/ml thrombin, as described above, then lipids extracted as described below. Exclusion criteria was a known sensitivity to aspirin.

### Lipid extraction

5 ng PGE_2_-d4, PGD_2_-d4, and di-14:0-phosphatidylethanolamine were added to samples before extraction as internal standards. Lipids were extracted by adding a solvent mixture [1 mol/L acetic acid, isopropyl alcohol, hexane (2:20:30, v/v/v)] to the sample at a ratio of 2.5 ml to 1 ml sample, vortexing, and then adding 2.5 ml of hexane (14). After vortexing and centrifugation, lipids were recovered in the upper hexane layer. The samples were then reextracted by addition of an equal volume of hexane. The combined hexane layers were dried and analyzed for free or esterified PGs using LC-MS/MS as below.

### Reverse-phase LC-MS/MS of free eicosanoids

Lipids were separated on a C18 Spherisorb ODS2, 5 μm, 150 × 4.6 mm column (Waters, Hertfordshire, UK) using a gradient of 50–90% B over 10 min (A, water:acetonitrile:acetic acid, 75:25:0.1; B, methanol:acetonitrile:acetic acid, 60:40:0.1) with a flow rate of 1 ml.min^−1^. Products were quantitated by LC-MS/MS electrospray ionization on a Sciex 4000 Q-Trap using parent-to-daughter transitions of *m/z* 351.2 [M-H]^-^ to *m/z* 271 for PGE_2_ and PGD_2_, *m/z* 355.2 to 275.3 for PGE_2_-d4 and PGD_2_-d4 with declustering potential of -55 and collision energies of -26 V. Products were identified and quantified using standards run in parallel under the same conditions. The following transitions were monitored: *m/z* 351.2 → 271 (PGE_2_ and PGD_2_), *m/z* 355.2 → 275.3 (PGE_2_-d4 and PGD_2_-d4).

### Reverse-phase LC-MS/MS of esterified eicosanoids

For analysis of PGE_2_/D_2_-phosphatidylethanolamines (PEs) in MRM mode, lipid extracts were separated by reverse-phase HPLC using a Luna 3 μm C18 (2) 150 mm × 2 mm column (Phenomenex) with a gradient of 50–100% B over 10 min followed by 30 min at 100% B (A, methanol/acetonitrile/water, 1 mmol/L ammonium acetate, at 60:20:20; B, methanol, 1 mM ammonium acetate) with flow rate 200 μl min ^–1^. MS was carried out using a Sciex 4000 Q-Trap, using declustering potential -140 V, collision energy -45 V. Lipids were monitored as parent *m/z* to daughter (*m/z* 271.2, as appropriate), with dwell time 200 ms.

### Phospholipase A_2_ hydrolysis

Platelet lipid extracts were dried using N_2_ then resuspended in 1 ml buffer [150mmol/L NaCl, 5mmol/L CaCl_2_, 10 mmol/L Tris (Trizma base), pH 8.9]. Two hundred micrograms snake venom phospholipase A_2_ (PLA_2_) from Sigma-Aldrich was added and incubated for 60 min at 37°C. Lipids were reextracted as described above using hexane:isopropanol:acetic acid.

### Oxidation of free and phospholipid-esterified arachidonate by purified/recombinant COX-1 and COX-2

Apo-COX-1 was purified from ram seminal vesicles and stored at 3.83 mg·ml^−1^ in 80 mMTris, pH 7.8, at −80°C (17, 18). Wild-type murine COX-2 (recombinant) was generated and purified as described (10.61 mg·ml^−1^) (19). Both enzymes were quantified using Bicinchoninic Acid (BCA) Protein Assay Kit (Thermo Fisher Scientific), according to manufacturer's instructions. For heme reconstitution, Apo-COX-1 or -2 (35 µg) was preincubated on ice for 20 min with 2 molar equivalents of hematin in phosphate buffer (100 mM potassium phosphate buffer, pH 7.4). Then, 3.5 μg of the reconstituted enzyme was added to 1 ml phosphate buffer and 500 µmol/L phenol and incubated for 3 min at 37°C in the presence of 150 µM arachidonate (AA, or AA-d8). In some experiments, the same amount of AA was replaced with 1-stearoly-2-arachidonyl-PE (SAPE). The reaction was stopped by addition of ice-cold lipid extraction solvent and immediate extraction of lipids, after addition of 5 ng each of PGE_2_-d4 and PGD_2_-d4 as internal standards, as described earlier. PGE_2_ and D_2_ were quantified by LC-MS/MS analysis as described earlier. In some experiments, 10 μmol/L of the metal chelator diethylenetriaminepentaacetic acid (DTPA) was added to the reaction just before the addition of holoCOX-1. The following transitions were monitored: *m/z* 634.4 → 227.2 (di-14:0-phosphatidylethanolamine), *m/z* 814.7 → 271.2 (PGE_2_/D_2_-PE), *m/z* 822.7 → 278.2 (PGE_2_/D_2_-PE-d8), *m/z* 351.2 → 271 (PGE_2_ and PGD_2_), *m/z* 359.2 → 278.2 (PGE_2_-d8 and PGD_2_-d8), *m/z* 355.2 → 275.3 (PGE_2_-d4 and PGD_2_-d4).

### Statistics

Data are representative of at least three separate donors with samples run in triplicate for each experiment. Data is expressed as mean ± SEM of three separate determinations. Statistical significance was assessed using an unpaired, two-tailed Student's *t*-test. Where the difference between more than two sets of data was analyzed, one-way ANOVA was used followed by Bonferroni multiple comparisons test as indicated in the legends. *P* < 0.05 was considered statistically significant.

## RESULTS

### Precursor scanning LC/MS/MS identifies esterified PGs in lipid extracts from thrombin-activated platelets

**Fig. 1.**
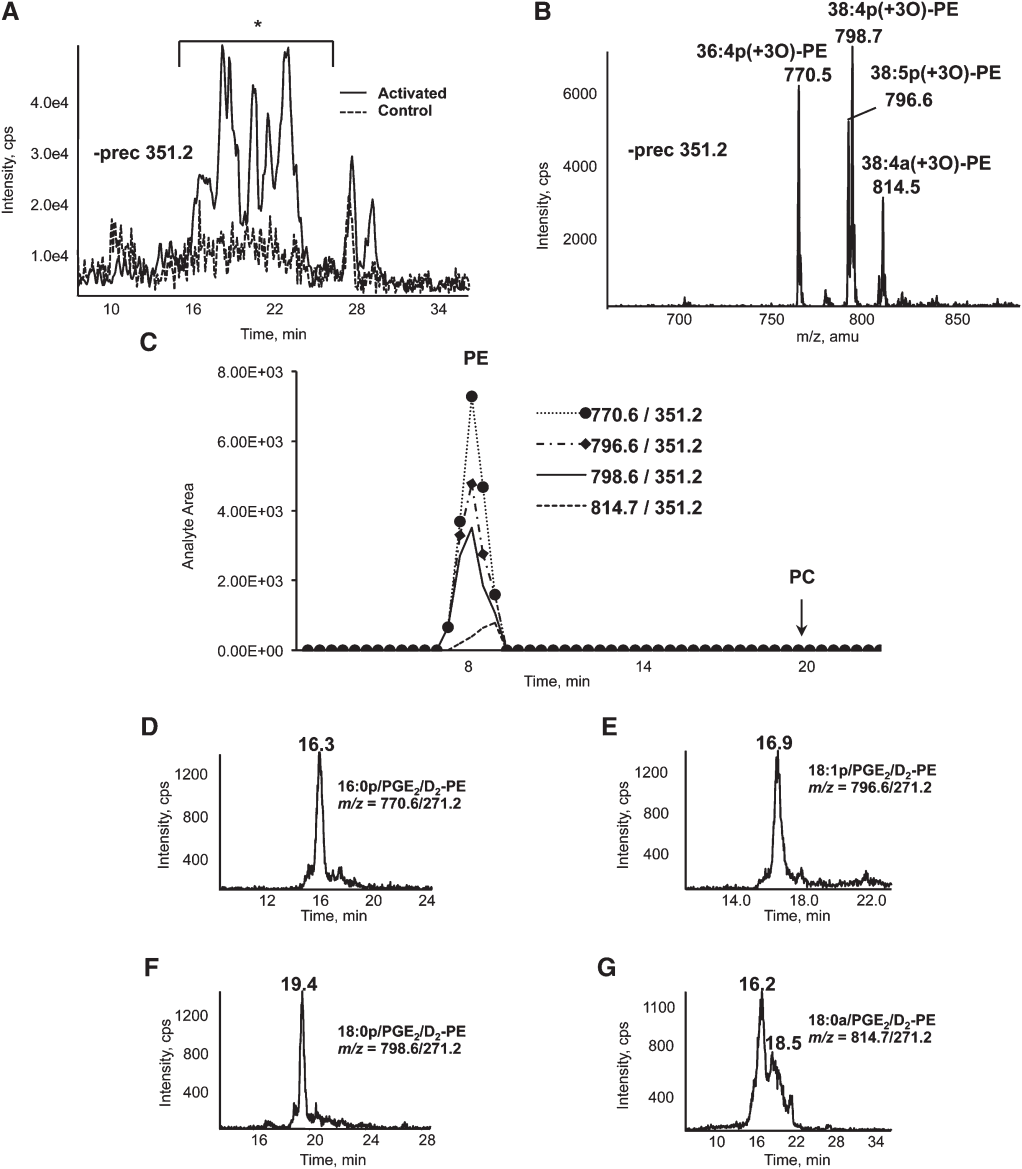
Identification of esterified PGs in human platelets and analysis of PGE_2_/D_2_-PE using LC/MS/MS*.* A: Precursor scanning demonstrates lipids with *m/z* 351.2 eluting during LC/MS/MS. Total lipid extracts from washed human platelets activated with 0.2 U/ml of thrombin for 30 min at 37°C were separated on the Q-Trap platform using LC/MS/MS as described in Materials and Methods, with online negative precursor scanning for *m/z* 351.2. *, region of LC trace where ions appear that are elevated by thrombin stimulation. Control, broken line. B: Identification of ions that generate *m/z* 351.2 daughter ions. Shown is a negative MS scan of region marked * in A. Scan shows ions eluting between 19 and 24 min. C: Characterizing phospholipid headgroups of esterified PL. Lipid extracts from thrombin-activated platelets were separated on normal-phase HPLC, as described in Materials and Methods, with fractions collected at 30 sec intervals. Twenty microliters of each fraction was analyzed specific parent → *m/z* 351.2 MRM transitions. PL class elution was determined using commercial phospholipid standards. Panels D–G: LC/MS/MS of PGE_2_/D_2_-PEs. Platelet lipid extracts were separated using LC/MS/MS as described in Materials and Methods and detected on the Q-Trap platform by parent → *m/z* 271.2.

**Scheme 1.**
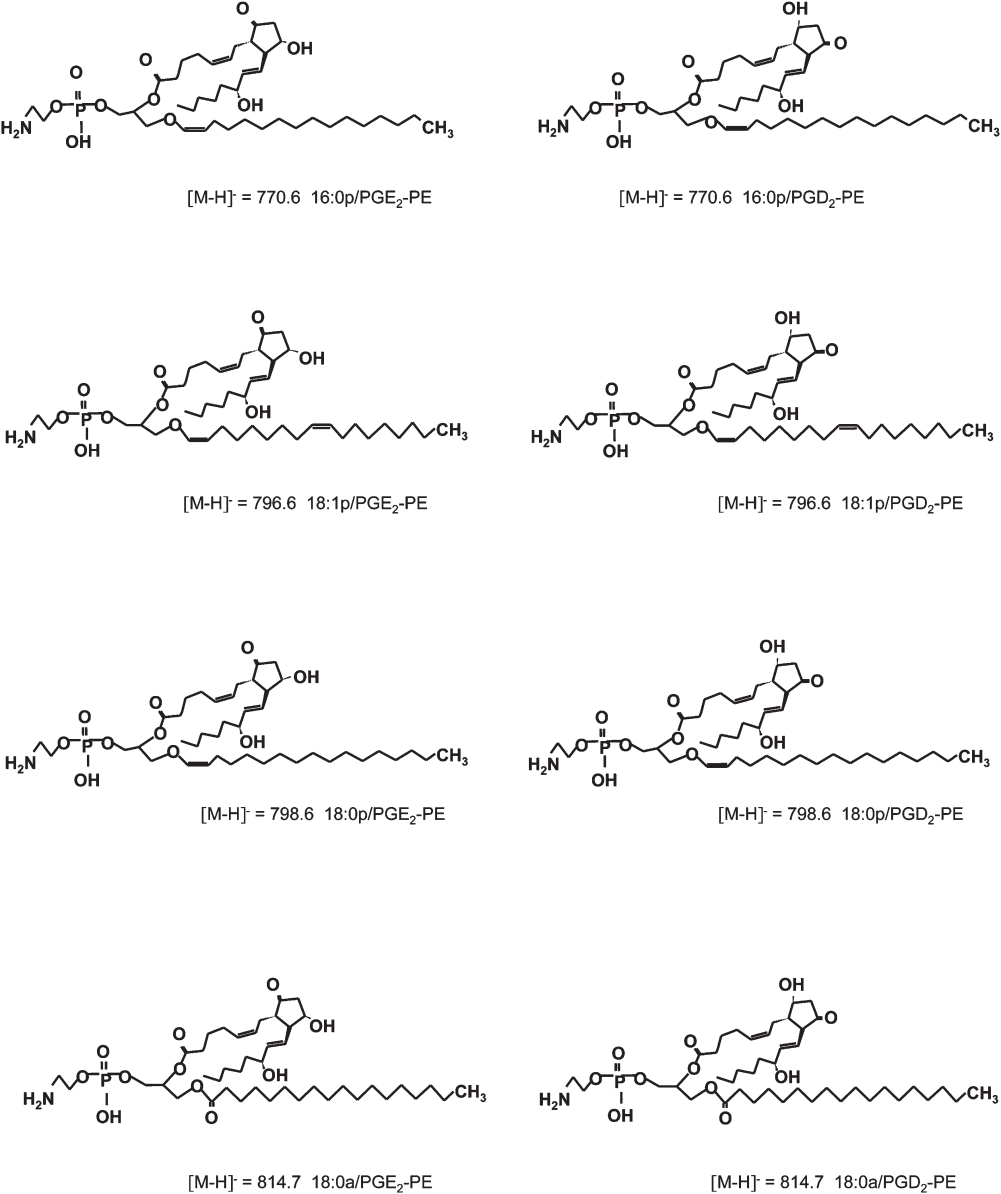
Structures of PGE_2_/D_2_-PE molecules identified in human platelets.

To identify PGs attached to larger functional groups, washed human platelets were activated using thrombin, then lipids extracted and analyzed using precursor-LC/MS/MS for *m/z* 351.2, the carboxylate anion of several PG species. Multiple ions eluted between 16 and 24 min that elevated on thrombin activation (**Fig. 1A**). Spectra acquired in this time window demonstrated four prominent ions at *m/z* 770, 796, 798, and 814, (Fig. 1B). These could represent either PE or phosphatidylcholine (PC), with PGs attached (20). Next, lipids were separated into PE and PC fractions using normal-phase HPLC and analyzed by flow injection LC/MS/MS, using parent-to-daughter transitions (parent *m/z* 770, 796, 798 and 814, daughter *m/z* 351.2). All four coeluted with the same retention time as a PE standard in the 7-9 min fraction (Fig. 1C). Thus, the ions are proposed as PEs containing 16:0p, 18:1p, 18:0p, and 18:0a at sn1 and a PG at sn2, and are termed prostaglandin-PEs (PG-PEs). Further analysis using MS/MS, MS^3^ and saponification followed by PG analysis demonstrated that one family of the lipids represents PGE_2_ and PGD_2_ esterified to PE (**Scheme 1**) (see supplementary data).

As we were unable to use *m/z* 351.2 for MRM detection of PGE_2_- and PGD_2_-PE (carboxylate daughter ion did not survive collision-induced-dissociation, as described in full in supplementary data), a method was established using *m/z* 271.1 as daughter ion (Fig. 1 D–G). However, because PGE_2_ and PGD_2_-PE lipids coelute on our system, in subsequent studies, each PGE_2_- and PGD_2_-PE pair (i.e., the same PE species) are reported as a single species.

### Human platelets acutely generate PGE_2_/D_2_-PEs on agonist activation that remain cell-associated

**Fig. 2.**
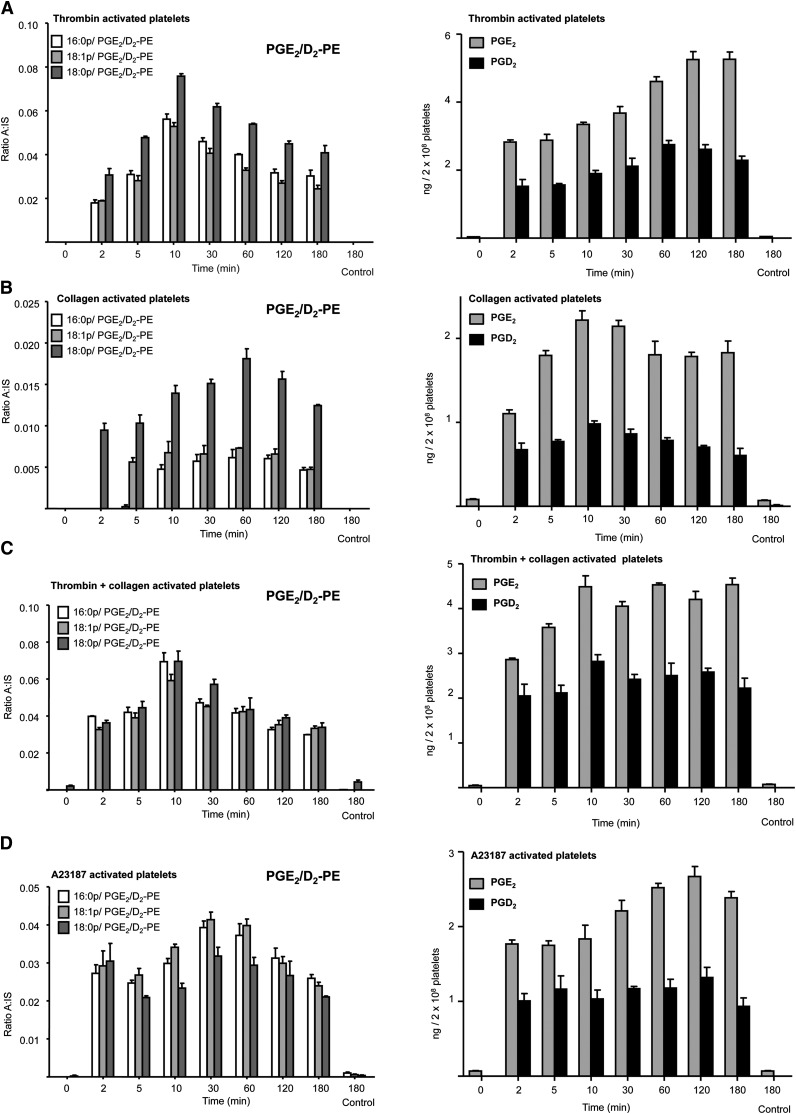
Generation of free and esterified PGs by agonist-activated platelets. Generation of PGE_2_/D_2_-PEs and free prostaglandins in response to pathophysiological agonists. Washed platelets were activated for varying times as shown, and lipids were extracted and analyzed using LC/MS/MS as described in Materials and Methods. Platelets were activated using 0.2 U/ml thrombin and PGE_2_/D_2_-PEs were determined (A). Platelets were activated using 10 µg/ml of collagen (B). Platelets were activated using 10 µg/ml of collagen and 0.2 U/ml of thrombin (C). Platelets were activated using 10 µmol/L A23187 (D). Levels of PGE_2_/D_2_-PE are expressed as ratio analyte to internal standard with experiments repeated at least three times on different donors (n = 3, mean ± SEM).

**Fig. 3.**
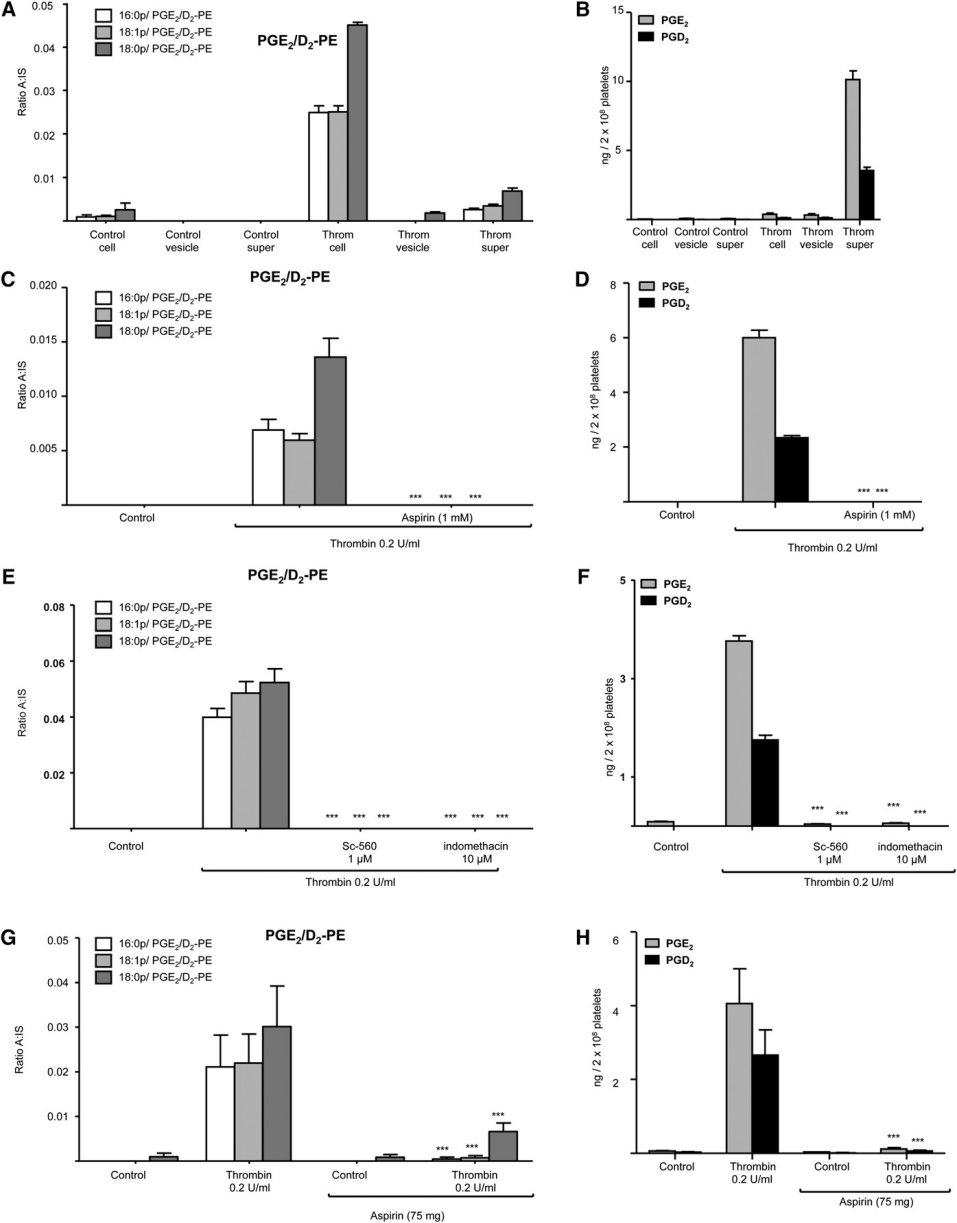
Esterified prostaglandins are retained by platelets while free PGE_2_ and PGD_2_ are primarily secreted, and generation of free and esterified PGs is sensitive to COX-1 inhibition in vitro and in vivo. A, B: Esterified PGs are retained by platelets. Washed human platelets were activated with 0.2 U/ml thrombin for 30 min before centrifugation at 970 *g*. The supernatant was centrifuged at 16,060 *g* to pellet microparticles before lipid extraction and analysis by LC/MS/MS. C, D: Esterified and free PGE_2_/D_2_ generation is sensitive to aspirin in vitro. Platelets were incubated for 15 min with 1 mM aspirin prior to thrombin activation (0.2 U/ml, 30 min) followed by lipid extraction and analysis of free and esterified PGE_2_/D_2_ using LC/MS/MS. E, F: Esterified and free PGE_2_/D_2_ generation is sensitive to COX inhibitors. Platelets were incubated for 10 min with 1 μM Sc-560 or 10 μM indomethacin prior to thrombin activation (0.2 U/ml for 30 min) followed by lipid extraction and analysis of free and esterified PGE_2_/D_2_ using LC/MS/MS. For all experiments described above, n = 3; mean ± SEM; data representative of three independent donors. *P < 0.05, **P < 0.01, and ***P < 0.001 versus control using ANOVA and Bonferroni post hoc test. G, H: Free and esterified PGE_2_/D_2_ formation in vivo is blocked by aspirin. Blood was obtained following a 14-day NSAID-free washout period for isolation of washed platelets and determination of free and esterified PGE_2_/D_2_ levels, as described in Materials and Methods, following activation using 0.2 U/ml thrombin for 30 min. Subjects then received 75 mg/day aspirin for 7 days before donation of a second blood sample and repeat determination of free and esterified PGE_2_/D_2_ levels. Data is representative of five independent donors (n = 5, mean ± SEM); *P < 0.05, **P < 0.01, and ***P < 0.001 versus control using ANOVA and Bonferroni post hoc test. Levels of PGE_2_/D_2_-PE are expressed as ratio analyte to internal standard. Levels of free PGE_2_ and PGD_2_ are expressed as ng/2 × 10^8^ platelets.

Temporal studies showed that PGE_2_/D_2_-PEs formed on activation with thrombin, collagen, or ionophore, similar to free PGE_2_ and PGD_2_ (**Fig. 2A–D**). While 16:0p-, 18:0p-, and 18:1p- species were always found, 18:0a/PGE_2_/D_2_-PE was sometimes under the limit of detection. Both free and esterified PGE_2_ and PGD_2_ were already detectable after 2 min activation. However, PGE_2_/D_2_-PEs levels peaked around 10 to 30 min before starting to decline, unlike free PGs, which remained stable or continue to increase up to 3 h after platelet activation (Fig. 2A–D). As standards are not yet available, we isolated PGE_2_/D_2_-PEs from platelets and quantified PG attached following hydrolysis and LC/MS/MS. Using this, we determined after 30 min of thrombin activation, mean values for esterified PGE_2_/D_2_-PE were 7.05 ± 0.7, 8.2 ± 0.9, 9.5 ± 0.5 and 3.3 ± 0.2 pg/2 × 10^8^ platelets (mean ± SEM, five genetically unrelated donors) for the 16:0p/, 18:1p/, 18:0p, and 18:0a/ forms, respectively, with a total for all four PE species of 28.1 ± 2.3 pg/2 × 10^8^ cells. PGE_2_/D_2_-PE was primarily retained (∼85%) by the platelets with small amounts appearing in either microparticles or supernatant (**Fig. 3A**). In contrast, ∼95% of free PGE_2_ and PGD_2_ was released into the supernatant (Fig. 3B).

### PGE_2_/D_2_-PE generation is blocked by COX-1 inhibition in vitro and in vivo

In vitro, several COX inhibitors including indomethacin, aspirin, and the COX-1 inhibitor, SC 560, completely inhibited PGE_2_/D_2_-PE formation as well as free PGE_2_/D_2_ (Fig. 3C–F). Furthermore, a 7 day supplementation with COX-1 selective low dose aspirin in vivo inhibited generation of both free and esterified PGE_2_/D_2_ by washed platelets in response to thrombin (Fig. 3G, H).

### PGE_2_/D_2_-PE forms in platelets via esterification of free PGE_2_/D_2_ into PE

**Fig. 4.**
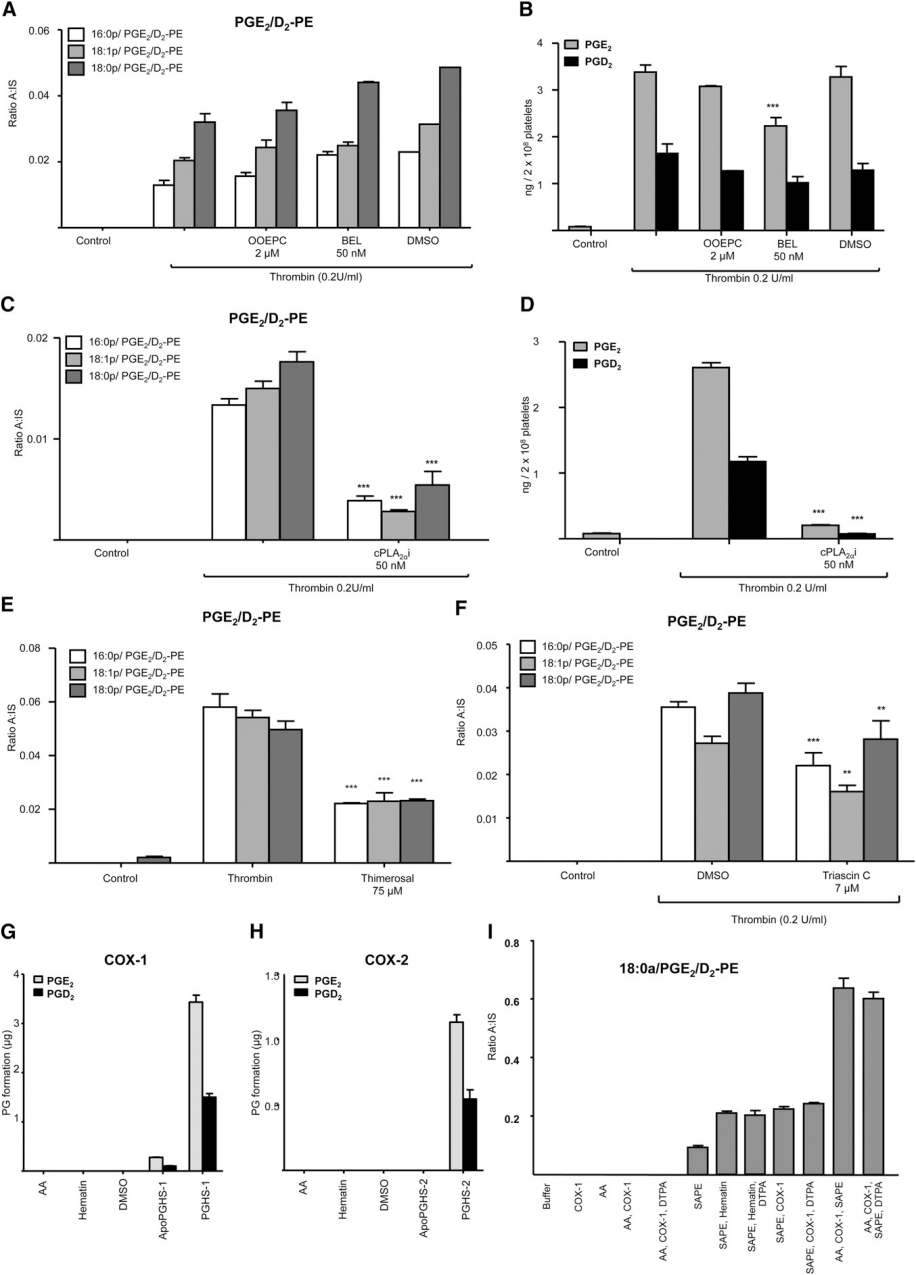
Generation of PGE_2_/D_2_-PEs requires cPLA_2_ and esterification of free eicosanoids. A-D: Generation of free and esterified PGE_2_/D_2_ requires cPLA_2_ but not other PLA_2_ isoforms*.* Washed human platelets were incubated for 10 min with each phospholipase A_2_ inhibitor prior to thrombin activation (0.2 U/ml for 30 min) followed by lipid extraction and analysis by LC/MS/MS. Inhibitors: cytosolic PLA_2_ (cPLA_2α_) inhibitor (cPLA_2α_i, 50 nmol/L), Ca^2+^-dependent secretory PLA_2_ (sPLA_2_) inhibitor (OOEPC2, 2 µmol/L), Ca^2+^-independent intracellular PLA_2_ (iPLA_2_) inhibitor (BEL, 50 nmol/L) or vehicle (DMSO, 0.5%). E, F: Inhibition of PGE_2_/D_2_-PE, by thimerosal or triascin C. Washed platelets were incubated for 30 min at 37°C with 75 µmol/L thimerosal or 7 mmol/L triascin C prior to thrombin activation (0.2 U/ml for 30 min) followed by lipid extraction and analysis using LC/MS/MS. For all experiments, n = 3; mean ± SEM; data are representative of three independent donors. *P < 0.05, **P < 0.01, and ***P < 0.001 versus control using ANOVA and Bonferroni post hoc test. G, H: COX isoforms generate a 2:1 ratio of PGE_2_:PGD_2_ in vitro. 3.5 μg of purified ovine COX-1 and 3.5 μg recombinant murine COX-2 were incubated at 37°C for 3 min with 150 µM of AA before lipid extraction and analysis using LC/MS/MS as described in Materials and Methods. PGE_2_ and PGD_2_ are expressed as micrograms/3.5 μg enzyme formed over 3 min (n = 3, mean ± SEM). I: PE is oxidized to PGE_2_/D_2_-PE during oxidation of AA by COX-1. Purified ovine Apo-PGHS-1 was reconstituted with hematin, ratio 2:1 (hematin: Apo-PGHS-1). 3.5 μg of purified ovine COX-1 was incubated at 37°C for 3 min with the following substrates: 150 µmol/L of AA; 150 µmol/L SAPE; liposomes containing AA and SAPE, in the presence or absence of 10 μM DTPA, before lipid extraction and analysis using LC/MS/MS as described in Materials and Methods. Levels of PGE_2_/D_2_-PE are expressed as ratio analyte to internal standard/3.5 μg enzyme generated over 3 min. PGE_2_ and PGD_2_ are expressed as micrograms/3.5 μg enzyme generated over 3 min (n = 3, mean ± SEM).

To determine whether PGE_2_/D_2_-PE generation by platelets is via direct oxidation of PE or fast esterification of newly formed eicosanoid, PLA_2_, and fatty acyl reacylation pathways were inhibited and the ability of purified and recombinant COX isoforms to oxidize AA-containing PE in vitro tested. Inclusion of several PLA_2_ inhibitors demonstrated requirement for cPLA_2_ (cPLA_2i_), but not iPLA_2_ BEL or sPLA_2_ OOEPC (**Fig. 4A, C**). Similar results were seen for free PG (Figs. 4B, D). Inhibition of fatty acylation using thimerosal or triascin C showed approximately 50% inhibition of PGE_2_/D_2_-PE generation (Fig. 4E, F). In separate experiments, platelets were supplemented with PGE_2_-d4 or AA-d8, at amounts similar to those generated during platelet activation, to determine whether exogenous lipids are incorporated into PE during the timescale of platelet activation. However, platelets never generated deuterated-PGE_2_/D_2_-PEs in our experiments (not shown).

Purified COX isomers generated PGE_2_ and D_2_ from AA with a 2:1 predominance of PGE_2_ over PGD_2_, due to decomposition of enzymatically-generated PGH_2_, similar to what is observed in platelets (Figs. 2, 4G, H). A small amount of PGE_2_/D_2_-PE was detected in 18:0a/20:4-PE (SAPE), even though it was a freshly opened vial, and this was increased by hematin (the COX-1 cofactor, added alone as control) through nonenzymatic oxidation (Fig. 4I). However, COX-1 did not elevate PGE_2_/D_2_-PE further, indicating it cannot directly oxidize SAPE. However, when SAPE was added during COX-1 oxidation of AA, a small formation of PGE_2_/D_2_-PEs was observed (Fig. 4I). Where AA-d8 was used instead of AA, deuterated forms of esterified PGs were not detected, indicating that esterified PGs originated directly via SAPE oxidation (not shown). Metal chelation (DTPA) did not inhibit formation, demonstrating that Fenton chemistry was not involved (Fig. 4I). Thus, it is likely that AA-derived radicals escaping from the COX-1 active site during turnover oxidize PE in a metal-independent manner generating isoprostane-PEs that include PGE_2_/D_2_-PEs. This may also occur in platelets as a minor pathway for esterified isoprostane formation, but it is unlikely to account for the majority of the PGE_2_/D_2_-PE formation as esterified isoprostanes were not detected in our experiments.

Collectively, the requirement for cPLA_2_ and COX-1 for platelet PGE_2_/D_2_-PE generation indicates that COX-1 oxidation of AA is required. Inhibition by triascin C and thimerosal suggest that PGE_2_/D_2_-PE generation in platelets occurs via reesterification of PGs. However, only endogenously generated PG is utilized, suggesting tight coupling between PGE_2_/D_2_ synthesis and incorporation into PE.

### Generation of PGE_2_/D_2_-PE requires PARs, intracellular calcium mobilization, src-tyrosine kinases, and MEK1, but not PI3-kinase, whereas PKC exerts negative feedback inhibition on generation

**Fig. 5.**
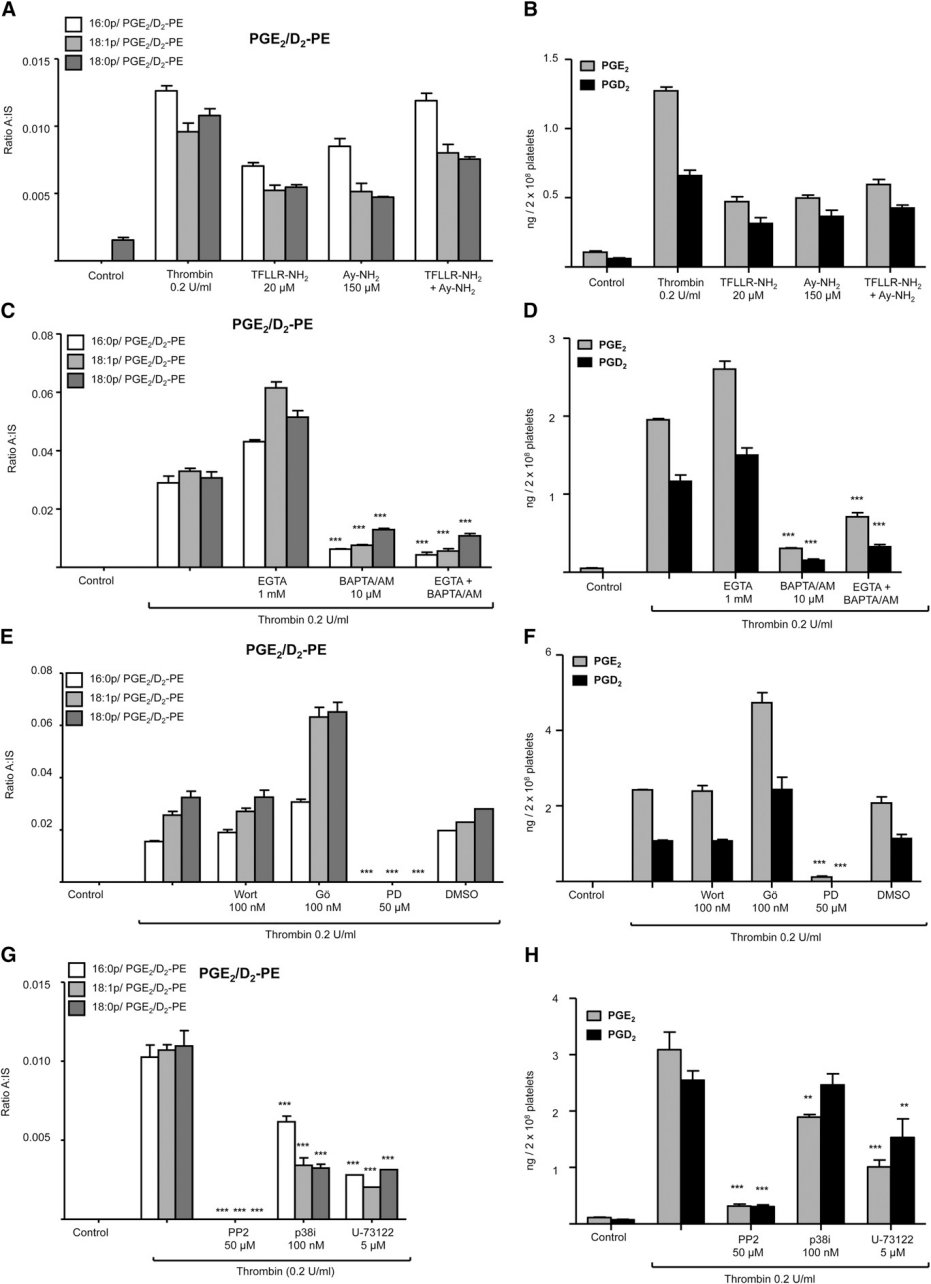
Elucidation of pathways involved in esterified and free PGE_2_/D_2_ generation by human platelets. A, B: Esterified and free PGE_2_/D_2_s are generated via PAR-1 and PAR-4 receptor stimulation. Washed platelets were activated with a PAR-1 agonist, TFLLR-NH_2_ (20 μmol/L), and/or a PAR-4 agonist AY-NH_2_ (150 μmol/L) for 30 min before lipid extraction and LC/MS/MS analysis as described in Materials and Methods. C, D: Cytosolic Ca^2+^ is required for thrombin-stimulated generation of esterified and free PGE_2_/D_2_. Washed platelets were incubated for 10 min with each inhibitor prior to thrombin activation (0.2 U/ml for 30 min) before lipid extraction and analysis as described in Materials and Methods. E-H: Phospholipase C, MEK1, p38 MAP kinases and *src* tyrosine kinases are required for thrombin-stimulated generation of PGE_2_/D_2_-PEs*.* Washed human platelets were incubated for 10 min with inhibitors prior to thrombin (0.2 U/ml, 30 min) followed by lipid extraction and analysis for free and esterified PGE_2_/D_2_. Inhibitors used are as follows: PI3 kinase (wortmannin, 100 nmol/L), protein kinase C (Gö 6850, 100 nmol/L), MEK1 (PD98059, 50 µmol/L), *src*-family tyrosine kinase (PP2, 50 µmol/L), p38 MAP kinase inhibitor (p38i, 100 nmol/L), and phospholipase C (U-73112, 5 µmol/L), or vehicle (DMSO, 0.5%). For all experiments, n = 3; mean ± SEM; data are representative of three independent donors. *P < 0.05, **P < 0.01, and ***P < 0.001 versus control using ANOVA and Bonferroni post hoc test. Levels of PGE_2_/D_2_-PE are expressed as ratio analyte to internal standard (n = 3, mean ± SEM).

**Scheme 2.**
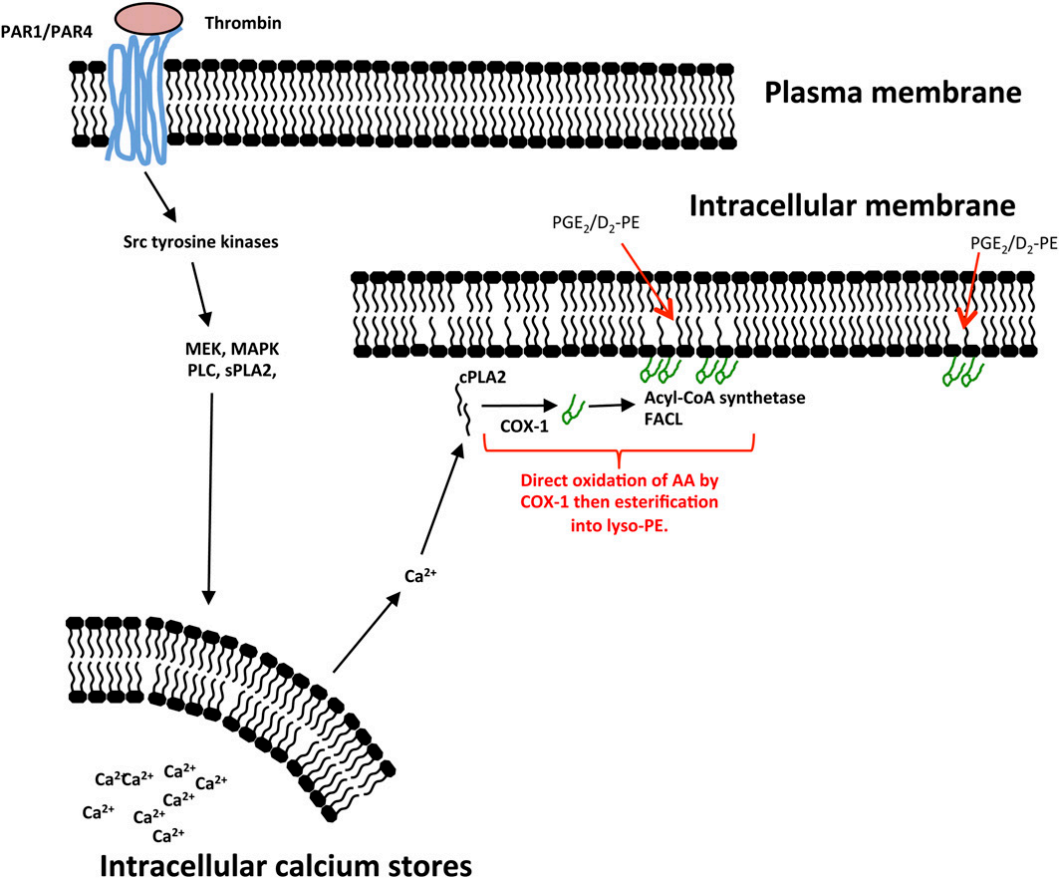
Proposed mechanisms for formation of PGE_2_/D_2_-PE by human platelet COX-1. First, AA is oxidized by COX-1, then esterified into PE. Formation of the lipids takes place in intracellular membranes where COX-1 and esterification enzymes are localized.

Thrombin activates platelets via PAR1 and PAR4. Peptide agonists for either receptor, TFLLR-NH_2_ (PAR1) or AY-NH_2_ (PAR4), stimulated generation of both esterified and free PGE_2_/D_2_ (**Fig. 5A, B**). Generation of PGE_2_/D_2_-PE and free PGE_2_/D_2_ in response to thrombin was inhibited by the cytosolic calcium chelator BAPTA/AM, but not by chelation of extracellular calcium by EGTA, implicating calcium mobilization from intracellular stores (Fig. 5C, D). Inhibition of PI3-kinase (wortmannin) was without effect, while blocking PKC (Gö 6850) significantly enhanced PGE_2_/D_2_-PE formation (Fig. 5E,F). This indicates that PKC exerts a negative feedback effect on free and esterified PGE_2_/D_2_ formation. Several agents effectively blocked generation including: U-73122, PD98059, PP2 and p38 MAP kinases inhibitor, implicating phospholipase C, MEK1, *src*-tyrosine kinases and p38 MAP kinases, respectively (Fig. 5E-H). Collectively, the data indicate a highly coordinated receptor and intracellular signaling pathway that is similar for both free and PE-esterified prostaglandins (**Scheme 2**).

## DISCUSSION

Herein, we showed that agonist-activated human platelets generate families of oxPLs via COX-1 that comprise esterified PGs, specifically PGE_2_ and PGD_2_ attached to PE. They form through a coordinated sequence of receptor and intracellular signaling pathways. We defined a new group of oxPLs and COX products, which form in platelets in response to pathophysiological agonists. OxPLs are families of bioactive lipids generated by both enzymatic and nonenzymatic pathways in vascular and immune cells (3, 7, 9–11, 13, 14). Up to now, it has not been considered that COX is a source of these; thus, this represents the first example of crosstalk between these two key lipid signaling pathways. It is also a new finding for COX-1, which has not been shown as a source of esterified PGs before.

PGE_2_/D_2_-PEs originated from COX-1 as their generation was sensitive to pharmacological inhibitors of this pathway both in vitro and in vivo. The primary product of COX-1, PGH_2_, is unstable in aqueous milieu, and in platelets, is either rapidly transformed to thromboxane A_2_ (TXA_2_) or undergoes rearrangement to PGE_2_ and D_2_ (21, 22). The PGD synthase inhibitor HQL-79 did not block PGD_2_ formation (data not shown). Platelets express mPGES-2 and cPGEs, which catalyze PGE_2_ generation from PGH_2_, while mPGEs-1 is not detectable (21). Because no selective mPGES-2 and cPGEs inhibitors were available, involvement of mPGES-2 and cPGEs could not be investigated. However, as free PGE_2_/D_2_ ratios are similar for both platelets and purified COXs, their generation in platelets from PGH_2_ prior to esterification is most likely nonenzymatic (Figs. 2, 4G, H).

The levels of these lipids are lower than for free PGs generated by platelets (28.1 ± 2.3 pg/2 × 10^8^ platelets). However, they are not secreted from the cells and thus maybe concentrated in intracellular membranes, leading to considerably higher local concentrations. Furthermore, bioactive phospholipids can signal at extremely low concentrations; for example, PAF that can cause life-threatening airway inflammation and inflammatory activation at only 10–1,000 pM concentrations (23).

PGE_2_/D_2_-PEs are generated within the first 2 min of platelet activation, similar to the timescale for generation of free PGE_2_/D_2_ (Fig. 2A–D). This indicates that formation is fast, coordinated, and a common event in response to several agonists. Their temporal generation (peaking around 10–30 min) could indicate further metabolism of esterified PGs through PLA_2_ hydrolysis or membrane remodeling pathways. The involvement of several signaling pathways indicates that this is a highly regulated event and further underscores their likely relevance to platelet biology. All these act upstream, stimulating cPLA2 and COX-1 and leading to generation of both free and esterified PGE_2_/D_2_ (Scheme 2). Partial inhibition using thimerosal or triascin C coupled with in vitro experiments showing that COX-1 cannot directly oxidize PE suggests that PGE_2_/D_2_-PEs form via reesterification of newly formed PGE_2_/D_2_ (Scheme 2). This idea is also supported by the absence of 8-iso-PGE_2_ and 11β-PGE_2_ in LC-MS/MS chromatograms of PGs hydrolyzed from platelet PE (supplementary Fig. III G). This mechanism is fully consistent with aspirin inhibition in vitro and in vivo. Due to the short timescales involved and the inability of PGE_2_-d4 to become esterified during platelet activation, it is likely that the proteins involved in formation and reesterification are closely associated such that AA hydrolysis, oxidation, and esterification are coordinated. We note that little is known regarding how oxidized fatty acids are esterified into phospholipids, and whether the enzymes involved display preferences for different fatty acids or eicosanoids. To address this, in separate experiements, rat liver microsomes were used as a model system to study PGE_2_ esterification into PE. However, although AA was efficiently esterified to either PE or PC, we were unable to detect PGE_2_-PE or -PC formation. We note that previous studies have shown that CoA independent transacylation reactions involved in ether lipid coupling are present in platelets but not rat liver microsomes, indicating that these systems likely contain very different complement of enzymes involved in fatty acid acylation (24).

The CoA-synthetases and lysophospholipid acyltransferases likely responsible for prostaglandin esterification are localized at the endoplasmic reticulum, mitochondrial membrane, and peroxisomal membranes (25). Furthermore, COX-1 is localized to dense tubule structures in platelets (26). Thus, PGE_2_/D_2_-PE generation may occur on intracellular membranes (Scheme 2). In this case, exogenously added PGE_2_ must enter the platelet in order to be esterified into PL. Thus, the lack of PGE_2_-d4 esterification might also be due to an inability of platelets to take up this lipid through the absence of prostaglandin transporters on the cell surface. To date, nothing is known regarding expression of these proteins by platelets nor how they utilize oxidized fatty acids as substrates (27).

PGE_2_/D_2_-PEs belong to a growing family of phospholipid-esterified eicosanoids that have been described in platelets and other circulating vascular cells over the last 5 years. Up to now, all were generated enzymatically by LOXs and in platelets include families of PE and PC that contain 12-hydroxyeicosatetraenoic acid (HETE) or 14-hydroxydocosahexaenoic acid (13, 14). Additional LOX-derived PE-esterified HETEs and keto-eicosatetraenoic acids have also been characterized in human neutrophils and monocytes (3, 9, 10). Platelet HETE-phospholipids are also generated following PAR receptor triggering, but the intracellular signaling cascade is partially distinct, involving sPLA_2_ and extracellular calcium, but not PLC. This likely reflects the different signaling pathways involved in 12-LOX versus COX-1 activation in platelets.

Unlike free eicosanoids, PGE_2_/D_2_-PEs remain membrane-bound, indicating that they are likely to act locally (Fig. 3A,B). This is similar to other enzymatically-generated oxPLs, such as HETE-phospholipids generated by LOXs, which regulate coagulation and immune cell signaling (3, 9–11, 14). Oxidized PLs contain polar groups that can protrude from the cell membrane surface. In the case of oxidized PCs, this has led to the “Lipid Whisker Hypothesis”, where the *sn2* fatty acid derivatives coat the outside of the cell and act as scavenger receptor ligands (28). Due to their shape and polarity, PGE_2_ and PGD_2_ attached to PE are also likely to protrude from the intracellular membrane surface, where they could interact with cytosolic proteins. Additionally, they could perturb membrane dynamics during platelet activation, through causing thinning or increasing water permeability, as shown to occur during chemical oxidation of membranes (29). This may play a role in vesiculation or degranulation, both events that involve significant membrane perturbations. Once sufficient quantities of these lipids can be generated, these ideas will all be tested in future studies.

In summary, COX-1 was found to generate a new family of oxPLs in platelets. The identification of these new metabolites opens the way for the study of how phospholipid-bound PGs may regulate membrane behavior during platelet function and whether these might be a distinct target for modulation in platelet-dependent pathologies.

## Supplementary Material

Supplemental Data
