# Supplementary material for: Human platelets generate phospholipid-esterified prostaglandins via cyclooxygenase-1 that are inhibited by low dose aspirin supplementation
Source: J Lipid Res. 2013 Nov;54(11):3085–97. doi: 10.1194/jlr.M041533 (PMC3793613; doi:10.1194/jlr.M041533)
Supplement: Supplemental Data [file supp_54_11_3085__index.html]

Human platelets generate phospholipid-esterified prostaglandins via cyclooxygenase-1 that are inhibited by low dose aspirin supplementation — Human platelets generate phospholipid-esterified prostaglandins via cyclooxygenase-1 that are inhibited by low dose aspirin supplementation — Supplemental Data 

# Human platelets generate phospholipid-esterified prostaglandins via cyclooxygenase-1 that are inhibited by low dose aspirin supplementation

## Supplemental Data

**Files in this Data Supplement:**

- Supplementary Data - Supplementary Data
