# Supplementary material for: Human platelets generate phospholipid-esterified prostaglandins via cyclooxygenase-1 that are inhibited by low dose aspirin supplementation
Source: J Lipid Res. 2013 Nov;54(11):3085–97. doi: 10.1194/jlr.M041533 (PMC3793613; doi:10.1194/jlr.M041533)

Supplementary Data.

Identification of one family of lipids as PGE_2 and PGD_2 esterified to PE.

Analysis of lipid extracts using multiple reaction monitoring (MRM), on a Sciex 4000 Q-Trap MS, with *m/z* 351.2 as daughter ion demonstrated two ions for each *m/z* (Supplementary Figure 1A-D). This suggests two distinct PG structures attached to PE. However, MS analysis monitoring their putative exact mass using Orbitrap FTMS indicated instead the presence of three lipids for each *m/z*, labeled "a-c" (Supplementary Figure 2A-D, solid line). Note that ions appear weaker, since the sensitivity of FTMS is lower, especially for the *m/z* 814 ion (Supplementary Figure 2D). Retention times using FTMS are altered due to different HPLC conditions, but subsequent MS/MS of all ions confirmed that the two lipids labeled "b" and "c" for each PE correspond with the pair of ions detected using tandem MS (data not shown), while peak "a" was not detected using MRM mode. To probe the reasons for the absence of peak "a" in MRM mode on the Q-Trap platform (Supplementary Figure 1 A-D), lipids were analyzed using the Orbitrap but isolating the parent in ITMS, fragmenting in CID then detecting the *m/z* 351.2 daughter ion using ITMS. This showed two peaks corresponding to "b" and "c" and only a small shoulder for "a" (Supplementary Figure 2A-D, dotted line). Thus, when analyzing using accurate mass, 3 esterified PGs are detected, but when analyzing by detection of the daughter ion of *m/z* 351.2, peak "a" disappears. This indicates that the *m/z* fragment 351.2 of lipid "a" is considerably more fragile than "b" or "c", and does not survive collision-induced dissociation (CID) during analysis. Thus, thrombin-activated platelets generate a total of 12 unique lipids, comprising four PE species with three structurally distinct PGs attached, designated "a-c".

Accurate mass MS/MS of the four PEs containing PG"a" was undertaken using the Orbitrap (with HCD fragmentation), during online separation of the lipids at the apex of elution for each lipid "a". Spectra were very similar, with several daughter ions characteristic of either PGE_2 or PGD_2 present in all four PEs (*m/z* 333, 271, 233 and 189) (Supplementary Figure 2E). Furthermore, all four showed isotope peaks characteristic of singly-charged species (Supplementary Figure 2E, insets). The spectrum of PGE_2 along with fragmentation pattern is shown for comparison (Supplementary Figure 3A). MS^3 analysis of these 4 PEs, in ITMS mode, further confirmed that the putative PGE_2/D_2 daughter ions originated from *m/z* 351.2 (Supplementary Figure 3B-E). The spectrum of *m/z* 814 showed a daughter ion at *m/z* 283, confirming the presence of 18:0a at sn1 (Supplementary Figure 2E). Additional ions are seen that arise from neutral loss of PG ketenes or PGs (e.g. at *m/z* 436 and 418 respectively, for *m/z* 770) (Supplementary Figure 2E). MS/MS of PEs containing PGs "b" or "c" showed that these contained PGs that are structurally distinct from "a" (not shown). However they did not match any known PGs as listed on LipidMaps, or The Human Metabolome Database. The structural characterization of these lipids will be undertaken separately.

Our MS/MS data suggested that peak "a" is either PGE_2, D_2, or isoprostanes, attached to PE, since these will have identical *m/z* and MS/MS fragmentation patterns. To confirm, platelets were activated using ionophore then lipid extracts analyzed for free and esterified PG isomers. Analysis of free eicosanoids demonstrated that platelets generated both PGE_2 and D_2, but not other E_2 isomers including 8-iso-PGE_2 and 11b-PGE_2 (Supplementary Figure 3F). Next, PG-PEs were purified from platelet lipid extracts by HPLC, hydrolyzed using PLA_2, and analyzed using LC/MS/MS. As with free eicosanoids, primarily PGE_2 and D_2 were detected (Supplementary Figure 3G). This indicates that peak "a" comprises a mix of PEs with primarily PGE_2 or D_2 attached (Scheme 1).

Supplementary Figure Legends.

Supplementary Figure 1.

Panels D-G. LC/MS/MS of the four putative prostaglandin-PE lipids shows the presence of two species for each m/z value. Platelet lipid extracts were separated and analysed using reverse phase LC/MS/MS as described in Experimental Procedures as parent → *m/z* 351.2.

Supplementary Figure 2. Structural characterization of esterified prostaglandins. *Panels A-D. High resolution LC/MS/MS indicates three distinct esterified PGs for each PL species.* Platelet lipid extracts were analysed using LC/MS/MS on the Orbitrap Platform, as indicated in Experimental Procedures, monitored either using accurate mass (solid line), or by fragmentation of parent *m/z* 351.2 (dashed line). *Panel E. MS/MS for peaks "a" demonstrate daughter ions characteristic for PGE_2/D_2.* Platelet lipid extracts were separated using LC/MS/MS on the Orbitrap platform as described, and MS/MS spectra acquired at the apex of elution of peak "a" for each *m/z* value, as shown. Inset shows the isotope pattern for each ion.

Supplementary Figure 3. Confirmation that peak "a" contains PGE_2 and PGD_2 esterified to PE. *Panel A. MS/MS spectrum of PGE_2.* An MS/MS spectrum of PGE_2 standard was acquired using the Q-Trap. Fragmentation shows the origin of daughter ions at *m/z* 271 and 189. *Panels B-E. MS^3 of parent PE lipids, by secondary fragmentation of daughter ion m/z 351.2, shows ions consistent with PGE_2/D_2.* Lipid extracts were analysed on the Orbitrap platform with ITMS detection, and CID fragmentation as described in Experimental Procedures. *Panels F,G. Analysis of free and esterified prostaglandins generated by human platelets shows both PGE_2 and PGD_2.* Platelet lipid extracts were analysed as described in

Experimental Procedures for free PGs (F). Free PGE_2 and PGD_2 isomer composition was determined based on comparison with prostaglandin standards. Esterified prostaglandins were purified using HPLC, hydrolyzed using PLA_2, then analysed for PGs using LC/MS/MS as described in Experimental Procedures (G).

Supplementary Figure 1

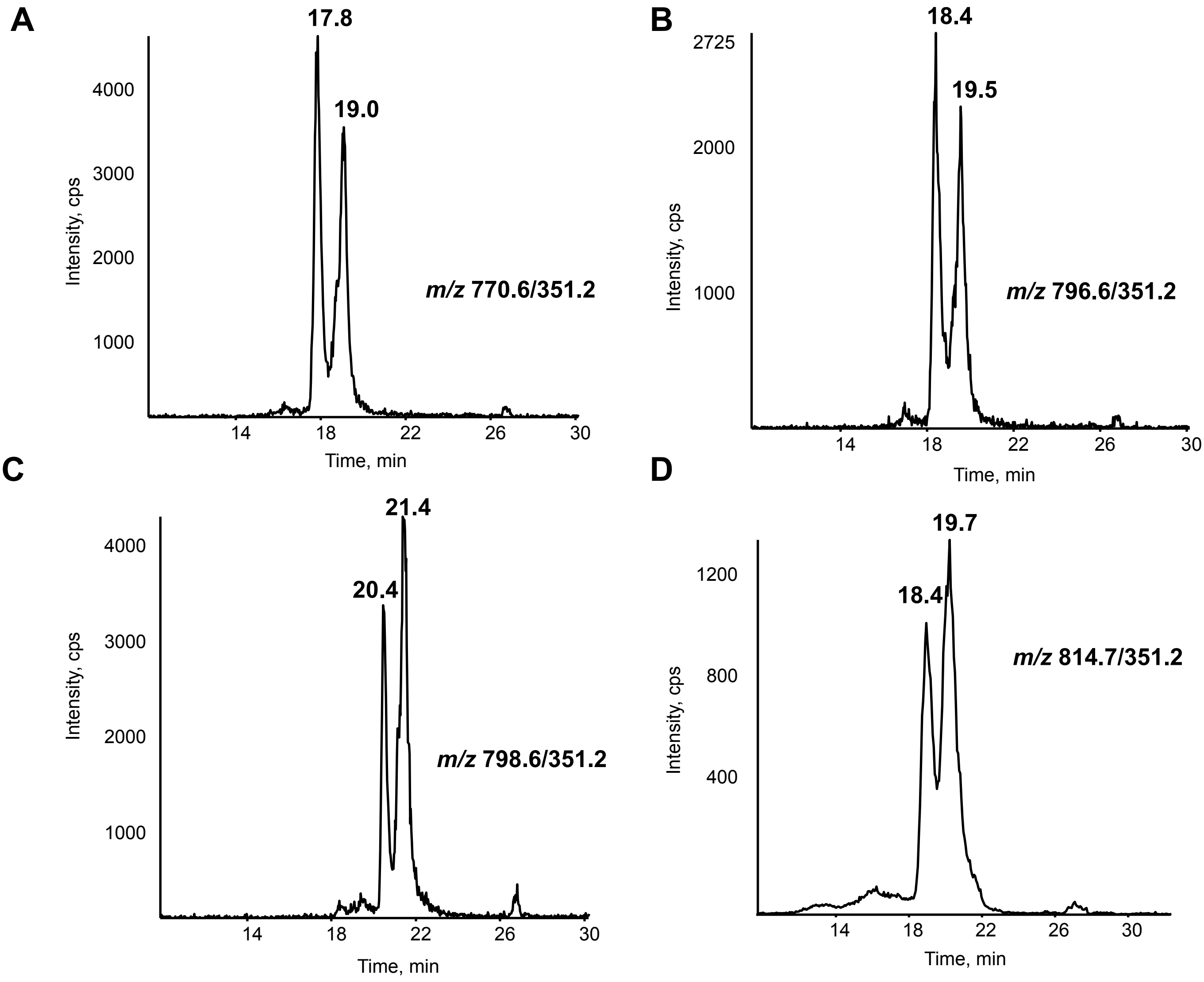

Supplementary Figure 2

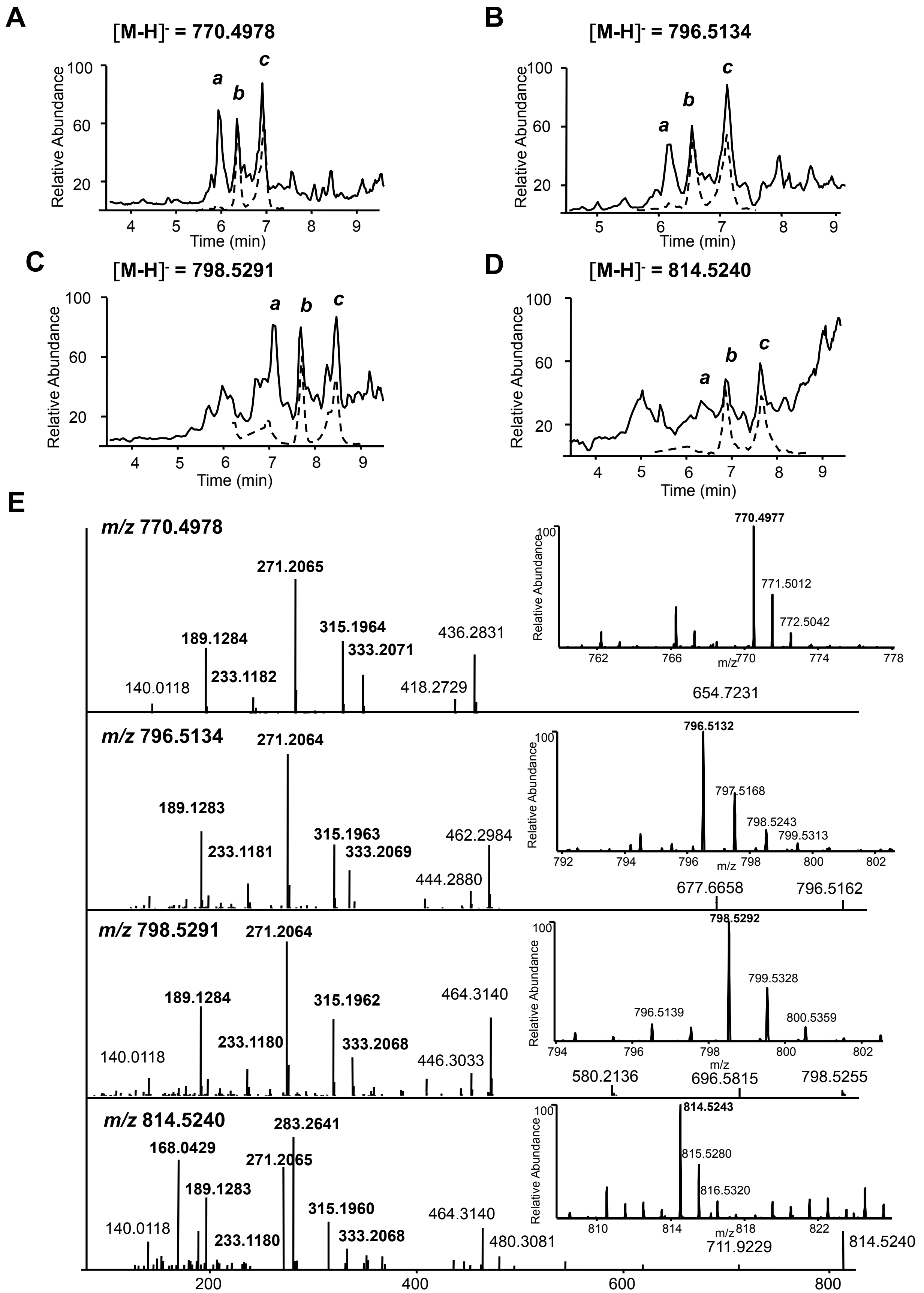

Supplementary Figure 3

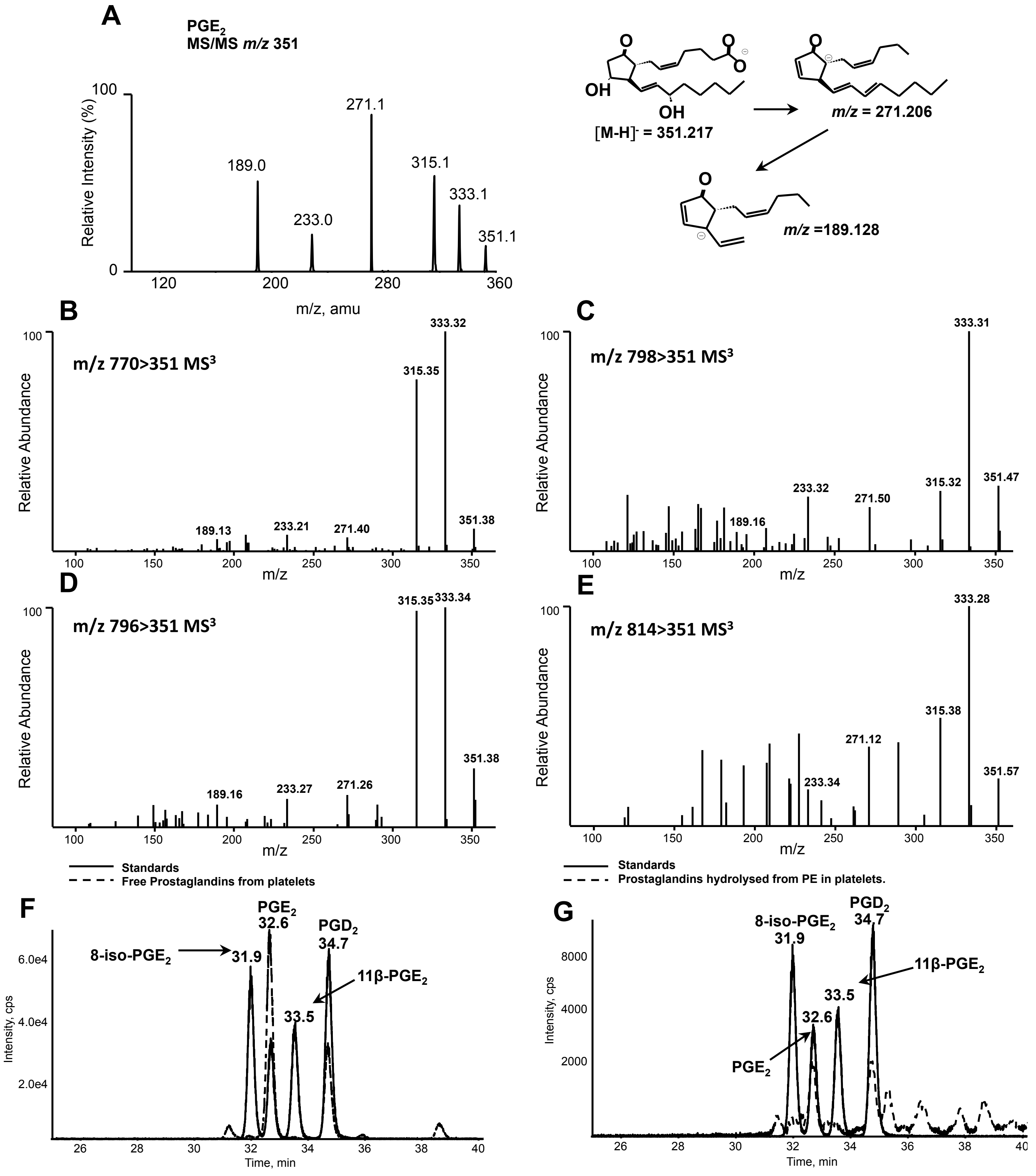

Supplement: Supplemental Data [file supp_M041533_jlr.M041533-1.pdf]
